# Investigation of the structure-odor relationship using a Transformer model

**DOI:** 10.1186/s13321-022-00671-y

**Published:** 2022-12-29

**Authors:** Xiaofan Zheng, Yoichi Tomiura, Kenshi Hayashi

**Affiliations:** 1 Graduate School of Information Science and Electrical Engineering, Department of Informatics, Kyushu University, Fukuoka, Japan; 2 Graduate School of Information Science and Electrical Engineering, Department of Electronics, Kyushu University, Fukuoka, Japan

**Keywords:** Molecular structure-odor relation, Transformer model, Odor descriptor

## Abstract

The relationships between molecular structures and their properties are subtle and complex, and the properties of odor are no exception. Molecules with similar structures, such as a molecule and its optical isomer, may have completely different odors, whereas molecules with completely distinct structures may have similar odors. Many works have attempted to explain the molecular structure-odor relationship from chemical and data-driven perspectives. The Transformer model is widely used in natural language processing and computer vision, and the attention mechanism included in the Transformer model can identify relationships between inputs and outputs. In this paper, we describe the construction of a Transformer model for predicting molecular properties and interpreting the prediction results. The SMILES data of 100,000 molecules are collected and used to predict the existence of molecular substructures, and our proposed model achieves an F1 value of 0.98. The attention matrix is visualized to investigate the substructure annotation performance of the attention mechanism, and we find that certain atoms in the target substructures are accurately annotated. Finally, we collect 4462 molecules and their odor descriptors and use the proposed model to infer 98 odor descriptors, obtaining an average F1 value of 0.33. For the 19 odor descriptors that achieved F1 values greater than 0.45, we also attempt to summarize the relationship between the molecular substructures and odor quality through the attention matrix.

## Introduction

Smell plays an important role in all aspects of life and is thus an important property of all compounds. The relationship between molecular structure and odor quality is an essential research topic. Studies on this relationship may lead to predictions of the odor of a molecule, odor synthesis, and even the artificial synthesis of molecules with specific odors. However, studying the odors of different substances is challenging. A previous study [1] showed that molecules with similar structures may have very different odors, while molecules with similar odors may have completely distinct structures. In addition to the subtle relationship between molecular structure and odor, aspects such as sex, age, and disease history can affect odor perception. Therefore, special training is required to label the odors of substances, which increases the difficulty of labeling the odors of chemical compounds. Thus, to date, the relationship between molecular structure and odor remains difficult to specify.

Machine learning has been applied in a wide range of fields, including physics and chemistry, and various molecular structure property prediction methods have been proposed [2–5]. These methods can be divided into feature-based methods and feature-free methods according to the type of data that are input into the model. Feature-based methods take the generated fixed molecular features (such as molecular fingerprints and molecular parameters) as model inputs and use various algorithms (e.g., random forest and support vector machines) to predict the molecular properties. Feature-free methods predict specific molecular properties by automatically extracting molecule features that are related to those properties using methods such as graph neural networks [6] or graph kernels [7]. In addition to predicting molecular properties such as water solubility and lipophilicity, feature-free methods use artificial neural networks to predict additional essential properties, such as the molecular energy, dipole moment and molecular dynamics [8, 9], allowing us to compute this information faster than using computational chemistry methods. In molecular property prediction, the interpretability of the model is particularly important [10], as model interpretability allows us to investigate the relationship between molecular structure and different properties at the molecular, atomic, and subatomic levels. Although feature-based methods use fixed features, the resulting model usually provides some interpretability. In contrast, feature-free methods flexibly extract features according to the properties to be predicted; however, the models are not often interpretable. Therefore, we aim to develop a feature-free method that allows interpretation of the extracted features.

At present, approximately 4000 odorants have been labeled with their corresponding odor. The smells of odorants have been labeled with odor descriptors (ODs), such as ‘sweet,’ ‘fruity,’ and ‘green.’ These data introduce the possibility of using data-driven approaches in molecular structure-odor studies. Several studies have used machine learning methods for OD prediction. For example, Keller et al. [11] used molecular parameters to predict the scores of 19 kinds of odors, achieving a correlation coefficient of 0.55. In contrast to most studies on OD prediction, this study attempted to predict scores corresponding to ODs through regression rather than classification, making it difficult to compare the results with those of the studies mentioned below. Shang et al. [12] predicted 10 ODs using molecular parameters, achieving an F1 value greater than 0.8. However, data augmentation was applied by synthesizing similar data points based on the original dataset before dividing the dataset into training and test sets. Therefore, the test set was essentially contaminated. Sanchez-Lengeling et al. [13] combined two datasets and predicted 138 ODs using a graph neural network (GNN) [3, 14], with the previous output layer applied to cluster the ODs. Although the average F1 value was 0.36, the clustering results showed that the outputs in the last layer were closer to each other when the corresponding molecules were labeled with ODs in similar categories. Chacko et al. [15] used the same dataset as Keller et al. [11] to predict the pleasantness and intensity of odors, as well as two ODs (sweet and musky). The corresponding F1 values of the two ODs on the test set were 0.84 and 0.69. The dataset used by Chacko et al. contained 480 samples, and the ratio of the training set to the test set was 9:1. Thus, the results may not be stable because of the small number of samples in the test dataset. Debnath and Nakamoto [16] predicted three ODs (fruity, green, and sweet) using the mass spectra of different molecules and achieved an average F1 value of 0.51.

In recent years, the Transformer model has been widely used in image processing [17, 18] and natural language processing [19, 20] because of its flexible attention mechanism. In addition to processing sentences and images, the Transformer model can take more flexible input forms (such as graphs) by using relative positional embedding [21, 22]. In terms of interpretability, the Transformer model results can naturally be interpreted according to its attention mechanism. Several Transformer models for molecular property prediction have been developed in recent years. Karpov et al. [23] used the SMILES data of molecules in the form of strings as the model input and predicted various molecular properties, such as the melting and boiling points. When molecules are represented in nonstring forms, the relative positional information between atoms must be used as one of the inputs to the model. Maziarka et al. [24, 25] predicted molecular properties by adding the relative positional information of the atoms to the attention matrix, and Maziarka et al. [26] used carefully designed functions to express the positional relationship between atoms based on Maziarka et al. [24]. Both of these works interpret the model by visualizing the attention mechanism in the encoder. Hutchinson et al. [27] and Thölke [28] predicted several more essential properties, such as the molecular energy, dipole moment, and molecular dynamics. They not only used carefully designed functions to express the positional relationship between atoms but also computed the outputs according to a more physical approach. For example, they predicted the atomic forces by computing the derivative of the predicted atomic energies with respect to the relative position.

In this research, we adopt a feature-free method and use the Transformer model to predict ODs. We first predict the existence of molecular substructures using the Transformer model and then evaluate the performance of the attention mechanism in terms of model interpretability by visualizing the attention matrix. Finally, we use the model to predict ODs and visualize the attention matrices.

The main contributions of this study can be summarized as follows:

- We finetune a Transformer model for predicting molecular properties and interpreting the results.
- Experiments are conducted to predict the existence of various substructures and to investigate the interpretability of the attention mechanism in the Transformer model.
- The developed Transformer model is used to predict ODs, and the attention matrix is visualized to identify OD structural features.

## Methods and experiment

### Model

The original Transformer model [19] was developed for machine translation and consists of an encoder and a decoder. Each layer in the encoder contains one attention module, which can be regarded as a self-attention mechanism through which each word in the input sentence interacts with related words. Each layer in the decoder contains two attention modules. The first attention module is also a self-attention mechanism that enables the word that is currently being translated to communicate with other translated words. The second attention module is used to obtain information about the source language for the current word.

A sentence is considered as a sequence of words. By adding position information as a positional embedding to the embedding of the input word, the original Transformer can consider the word order. Molecules are three-dimensional (3D) structures that are composed of atoms. The relationship between atoms in a molecule cannot be represented by the positional embeddings used in the original Transformer because the bonds between atoms must be represented.

**Fig. 1:**
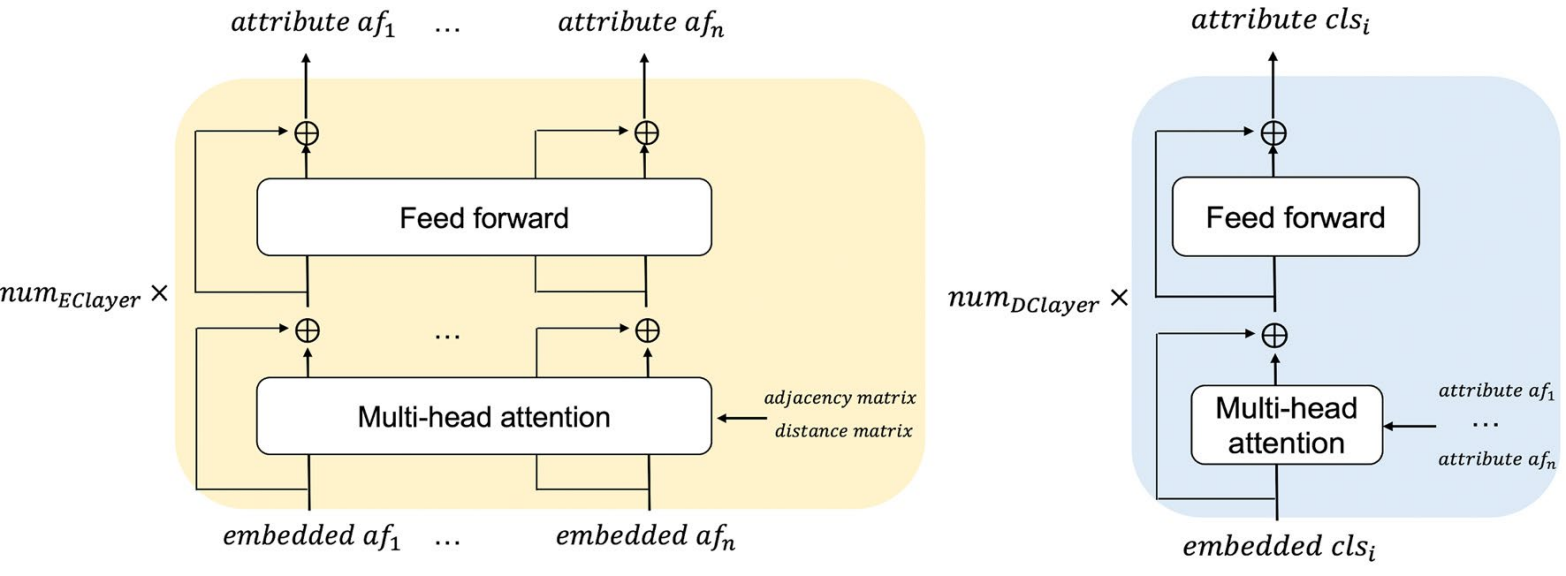
The details of the encoder and decoder-like modules are shown on the left and right. Encoder: The inputs to the encoder are embedded atomic features (*embedded af*), the adjacency matrix, and the distance matrix. The outputs of the encoder are atomic attributes (*attribute af*). Decoder-like module: the inputs are vectors (*embedded cls*) corresponding to different outputs from the encoder

The Molecular Attention Transformer (MAT) model [24] was developed to predict molecular properties such as water solubility and blood-brain barrier penetration. The MAT model provides a creative solution for identifying the relationship between atoms. As shown on the left side of Fig. 1, the MAT model replaces positional embedding by adding adjacency and distance matrices to the attention matrix. The attention mechanism in the MAT model is formulated as

1 $$\begin{aligned} Attention = \left( \lambda _{1}softmax\left( \frac{QK^{T}}{\sqrt{d_{k}}}\right) +\lambda _{2}g(D)+\lambda _{3}A\right) V, \end{aligned}$$

where $\lambda _{1}$, $\lambda _{2}$, and $\lambda _{3}$ are hyperparameters; *Q*, *K*, and *V* are the query matrix, key matrix, and value matrix (as in the original Transformer); *D* and *A* are the distance matrix and adjacency matrix, respectively; and $g(d)=exp(-d)$ is an elementwise function.

In this study, we propose a model based on the original Transformer and MAT models. We do not use more complex interatomic distance formulas or more distant neighborhood information as the direct inputs to the model, as used by Maziarka et al. [26]. Instead, we expect the model to automatically learn more complex distance and adjacency relationships through multiple heads and multiple encoder layers. The key features of the proposed model can be summarized as follows: (1) changes the attention calculation; (2) adds a decoder-like structure to the model to improve interpretability; and (3) introduces a contrastive loss function to the model.

In the MAT model, attention is calculated by summing the inner product between the atom attributes, adjacency matrix, and distance matrix. According to Eq. (1), if the inner product between two atoms is large and these two atoms are far away from each other, information is exchanged between the two atoms, which is unreasonable. In this study, we change Eq. (1) to the following equation:

2 $$\begin{aligned} \begin{aligned} Attention&= \left( softmax\left( \frac{Q_{adj}K_{adj}^{T}}{\sqrt{d_{k}}}\right) \odot A\right) V_{adj} \\&\quad +\left( softmax\left( \frac{Q_{dist}K_{dist}^{T}}{\sqrt{d_{k}}}\right) \odot g(D)\right) V_{dist}, \end{aligned} \end{aligned}$$

$Q_{adj}$ is obtained by a linear transformation of the input to the encoder layer ($Q_{adj} = X W^{Q}_{adj}$, where *X* is the input to the encoder layer and $W^{Q}_{adj}$ is a learnable parameter); $Q_{dist}$, $K_{adj}$, $K_{dist}$, $V_{adj}$, and $V_{dist}$ can be obtained in the same way. On the basis of Eq. (2), message passing between two atoms based on their inner product value occurs only when the atoms are connected by a chemical bond or the atoms are close to each other.

**Fig. 2:**
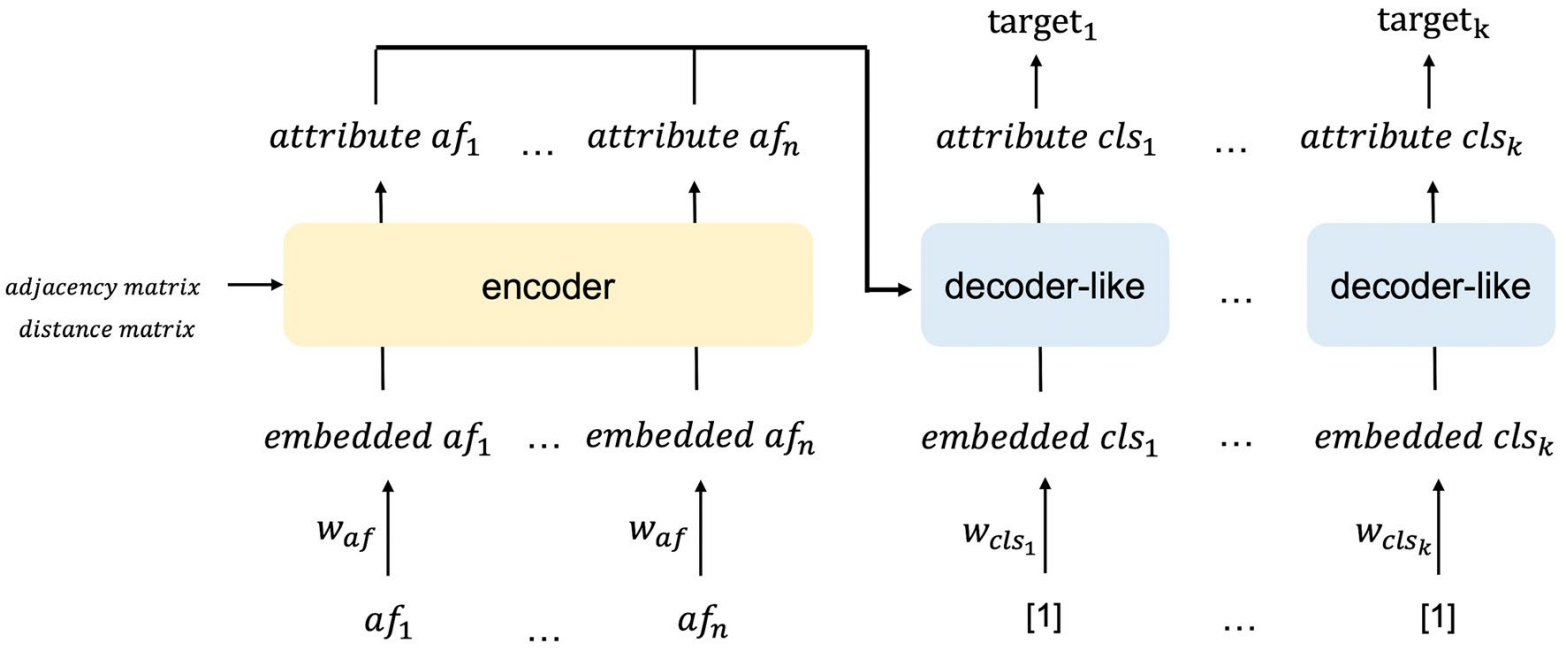
Proposed model: the inputs are atomic features (*af*), there are multiple decoder-like modules for different targets, and there is a single fully connected layer between *attribute cls* and the targets

In the MAT model, the output of the encoder is directly passed through a pooling layer before the molecular properties are predicted by the fully connected layers. We add a decoder-like module, similar to the original Transformer, to visualize the relationship between the atoms and outputs. The proposed model is shown in Fig. 2. In natural language, the words in a sentence are related to each other. However, in most cases, ODs are not necessarily related to each other. Therefore, we use a decoder-like module, namely, the Transformer decoder without the self-attention mechanism, as shown on the right side of Fig. 1. The output of the transformer encoder is transmitted to this decoder-like module. As shown in Fig. 2, the input to the decoder-like module is $embedded\,cls_{i}$, which is obtained by passing a scalar of value 1 through a single fully connected network. Thus, $embedded\,cls_{i}$ is a learnable input for $\mathrm target_{i}$. The attention in the decoder-like module is computed by considering $embedded\,cls_{i}$ as the query and the outputs of the encoder as the key and value. This attention mechanism in the decoder-like module is expected to obtain better predictions by emphasizing atoms that are related to the molecular properties, thereby enabling the visualization of important substructures that affect the prediction results.

The contrastive loss function has been widely used in self-supervised learning in recent years [29, 30]. The application of the contrastive loss to supervised learning [31] can also improve model performance. We directly apply this contrastive loss to our model, and the definition of the loss function is shown in Eq. (3).

3 $$\begin{aligned} L_{contrastive}=\sum _{i\in batch}L_{i}=\sum _{i\in batch}\frac{-1}{|P(i)|}\sum _{p\in P(i)}\log \frac{exp(\varvec{z_{i}}\varvec{z_{p}}/\tau )}{\sum _{a\in A(i)}exp(\varvec{z_{i}}\varvec{z_{a}}/\tau )} \end{aligned}$$

*P*(*i*) is a set that includes all samples whose labels are the same as sample *i*, $|\bullet |$ is a function that counts the number of elements in a set, $z_{i}$ is a feature vector with unit length corresponding to sample *i*, *A*(*i*) is a set that includes all samples in the batch except sample *i*, and $\tau$ is a hyperparameter. The contrastive loss function brings feature vectors of samples with the same label closer while separating feature vectors of samples with different labels.

### Experiment

We conducted two experiments in this research. The first experiment aimed to predict whether the input molecule has some specific substructure using our proposed Transformer model. The second experiment predicted ODs using the proposed Transformer model.

**Table 1:** Atomic features

	Values
Atomic identity	C, O, S, N, Cl, Na, P, F, Mg, I, Br, Zn, Fe, As, Ca, B, Si, K, Co, Cr, H, Al, others
Number of heavy neighbors	0, 1, 2, 3, 4, other
Number of hydrogen neighbors	0, 1, 2, 3, 4, other
Is aromatic	0, 1
Is in ring	0, 1
Hybridization type	S, SP, SP2, SP3, SP3D, SP3D2, unspecified, other
Chirality	CW, CCW, unspecified, other
Formal charge	0, -1, 1, -2, 2, 3, 4, other
Explicit valence	0, 1, 2, 3, 4, 5, 6, 7, other
Implicit valence	0, 1, 2, 3, other

We used two different datasets for these two experiments. We collected SMILES data for 100,000 molecules from ChEMBL [32] for the substructure predictions. The ChEMBL database is a bioactive dataset covering more than 2 million compounds, which ensures that we have sufficient data for substructure prediction. For the OD prediction experiment, we collected 4,240 odorants and their corresponding ODs from TheGoodScentsCompany [33]. Among the datasets that provide OD labels, TheGoodScentsCompany provides more data that is easier to obtain. In addition to odorants, we collected 222 molecules that were annotated as odorless from TheGoodScentsCompany. RDKit with default settings was used to compute the atomic properties, adjacency matrices, and distance matrices of all molecules. For both datasets, we removed molecules for which the distance matrix could not be calculated and molecules with more than 60 atoms. Finally, 98,324 and 4,365 samples were used in the substructure prediction and OD prediction experiments, respectively. The model inputs were the atomic properties presented in Table 1. The code used for the experiments can be found at [34].

#### Substructure prediction

**Fig. 3:**
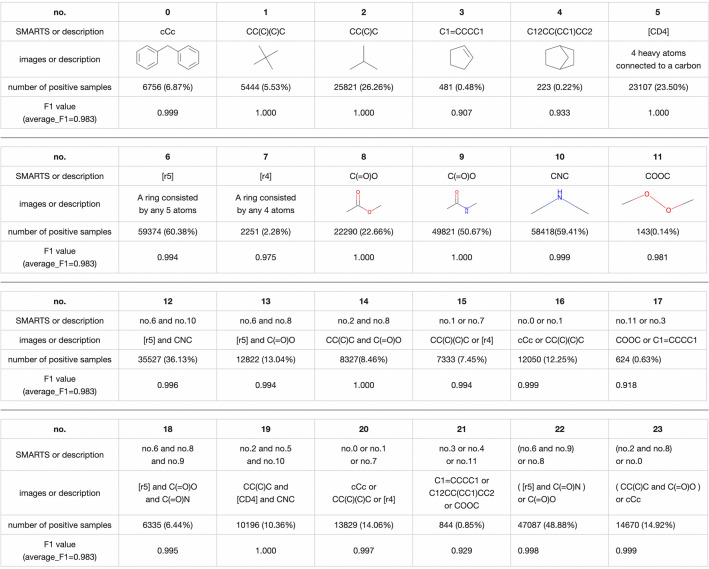
Details of the substructure experiment

The purpose of this experiment was to test the performance of the Transformer model in predicting the existence of substructures and to investigate the interpretation ability of the model by visualizing the attention mechanism in the decoder-like module. We designed 24 substructures and combinations of multiple substructures and predicted these substructures with our proposed model. Fig. 3 shows descriptions of the 24 substructures and the corresponding number of positive samples. Substructures No. 0 to No. 11 are individual substructures, and substructures No. 12 to No. 23 are combinations of 2 or 3 substructures.

**Table 2:** Hyperparameters used in the substructure prediction experiment

	Setting 1	Setting 2	Setting 3	Setting 4
Number of heads	12	12	12	12
Dimension of a single head	15	15	15	15
Number of encoder layers	6	6	6	6
Number of decoder layers	1	2	3	4
Average F1 value	0.985	0.970	0.976	0.966

The 98,324 samples were divided into training and test sets at a ratio of 5:1. Because we have sufficient data in this experiment and predicting the existence of substructures is a relatively simple task, we did not consider a wide range of hyperparameter settings. The hyperparameter settings examined in this experiment are listed in Table 2. In addition to the parameters listed in Table 2, similar to the original Transformer, each encoder and decoder layer includes an attention module and a two-layer pointwise feedforward network with the same number of units as the dimension of the atomic attributes, both of which end with a dropout layer with a rate of 0.1. Except for the layers used to convert the Q, K, and V matrices, which do not use the activation function, and the final output layer, which uses the sigmoid activation function, the rest of the fully connected layers use ReLU as the activation function. The learning rate was set to 7e-5 in this experiment.

**Fig. 4:**
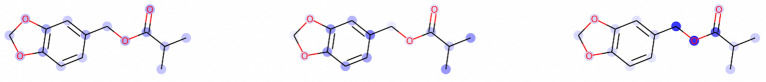
Visualization of a sample result

In the multihead attention mechanism, each decoder layer should contain multiple attention matrices. Hence, our visualization results are the sum of the attention matrices of multiple heads. An example of visualizing the attention in the decoder-like module is shown in Fig. 4. This figure shows the visualization results of a molecule predicted by a model with three decoder-like layers. The three subfigures correspond to the attention mechanisms of the three decoder-like layers. The values in the attention matrices are indicated by the shade of the blue circle covering each atom, with light blue indicating a small value and dark blue indicating a large value. (In the default RDKit settings, the atoms are identified by different colors depending on the atomic number; for example, ‘O,’ ‘N,’ and ‘S’ are written in red, blue, and yellow, respectively. Thus, the red character ‘O’ in Fig. 4 is not relevant to the attention.)

In addition to visualizing the attention matrices, we attempted to quantify the performance of the attention mechanism in terms of identifying atoms related to predictions. For one molecule, we picked out the atoms contained in a target substructure and summed the corresponding attention values according to

4 $$\begin{aligned} \sum _{a\in T_{i}}attn^{i}(a), \end{aligned}$$

where $T_{i}$ is the set of atoms contained in the *i*-th target and $attn^i(a)$ denotes the attention value of atom *a* when predicting the *i*-th target. (Note that each attention matrix sums to 1 for all atoms; thus, the value of Eq. (4) should be between 0 and 1, with larger values indicating that the attention mechanism better identifies atoms related to the target substructure.) In later experiments, we visualized the sum of the attention matrices of all decoder layers and all heads. Therefore, the sum of the attention values of all atoms in a molecule is the product of the number of heads ($n_h$) and the number of decoder layers ($n_{dc}$). The performance of the attention mechanism in terms of identifying related atoms is evaluated as

5 $$\begin{aligned} \frac{\sum _{a\in T_{i}}attn^{i}(a)}{n_{h}\cdot n_{dc}} \end{aligned}$$

To compute the sum of the attention values of the target atoms, we calculated the variance of the attention values of the target atoms. A large variance indicates that the attention mechanism tends to identify only some of the target atoms, while a small variance denotes that the attention mechanism uniformly identifies the target atoms. For each molecule, the variance is calculated as

6 $$\begin{aligned} \frac{1}{|T_{i}|}\sum _{a\in T_{i}}\left( attn^{i}(a)-\frac{\sum _{a^{'}\in T_{i}}attn^{i}(a^{'})}{|T_{i}|\cdot n_{h}\cdot n_{dc}} \right) ^2, \end{aligned}$$

where $|T_{i}|$ is the number of atoms that belong to the target.

#### OD prediction

The number of positive samples of most ODs is less than 1/10 of the dataset, with ‘fruity’ having the most positive samples (1334). Moreover, 10 ODs have more than 400 positive samples, namely, ‘fruity’, ‘sweet’, ‘green’, ‘floral’, ‘woody’, ‘herbaceous’, ‘fresh’, ‘fatty’, ‘spicy’, and ‘waxy’. Among the ODs, 98 ODs had more than 50 positive samples. We predicted these 98 ODs in this experiment.

**Fig. 5:**
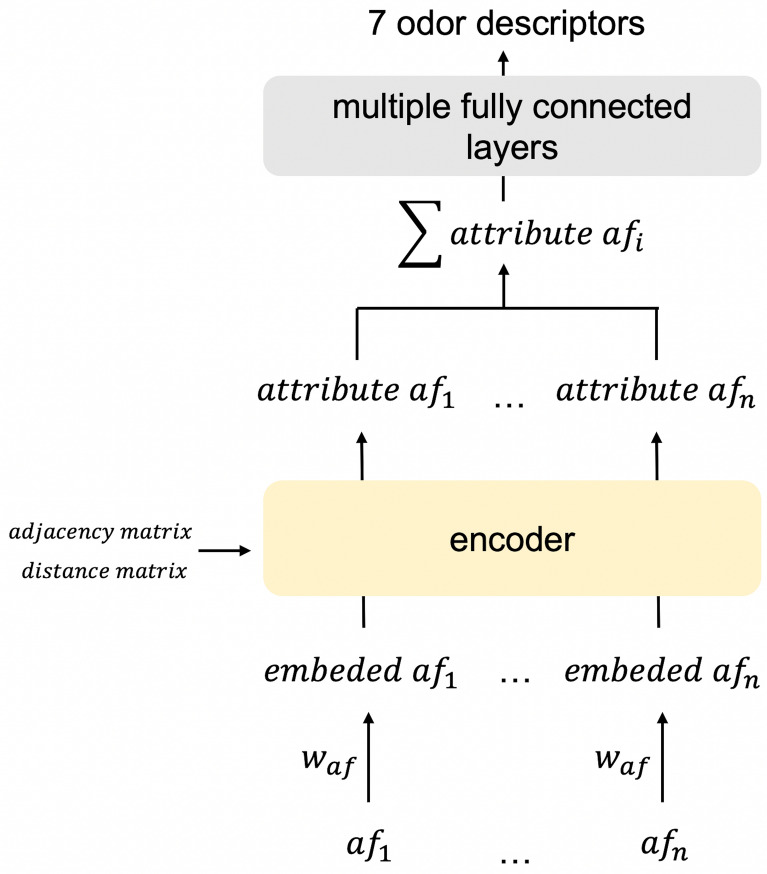
Simplified decoder model

**Table 3:** Hyperparameters used in the OD prediction experiment

	Values	Optimal OD prediction setting
Number of heads	6, 8, 10, 12	8
Dimension of a single head	30, 50	30
Number of encoder layers	5, 6, 7, 8	7
Number of decoder layers	1, 2	2
$\tau$ in contrastive loss	0.3, 0.7, 1.0	0.7

In the OD prediction experiment, we compared the following six models. Proposed model: a model with the attention calculated using (2); MAT-attn: a model with the attention calculated using Eq. (1); ADJ-only: a model with the attention calculated using Eq. (7); DIST-only: a model with the attention calculated using Eq. (8); Simplified decoder: the model shown in Fig. 5, which was created based on the proposed model by simplifying the decoder-like module to a sum pooling layer; MAT-model: the original MAT model. ADJ-only and DIST-only were used to investigate the role of the adjacency and distance matrices. The simplified decoder model was used to investigate the effect of the decoder-like module.

7 $$\begin{aligned} Attention= & {} \left( softmax\left( \frac{QK^{T}}{\sqrt{d_{k}}}\right) \odot A\right) V \end{aligned}$$

8 $$\begin{aligned} Attention= & {} \left( softmax\left( \frac{QK^{T}}{\sqrt{d_{k}}}\right) \odot g(D)\right) V \end{aligned}$$

The ratio of the training set to the test set was fixed at 5:1. The hyperparameter settings used in this experiment are listed in Table 3. In this experiment, class weights were used in the loss function, e.g., for each OD, the weight of each negative sample was 1, and the weight of a positive sample was equal to (number of all samples - number of positive samples)/number of positive samples.

## Results and discussion

### Substructure prediction results

The best average F1 value for the 24 substructure prediction experiment was 0.983, which was achieved with 12 15-dimensional heads, six encoder layers, and one decoder layer. The individual F1 values for the 24 substructures were all greater than 0.9, as shown in Fig. 3. The average F1 values for the other hyperparameter settings are listed in Table 2 and are generally very similar to one another. In summary, our proposed Transformer model can detect the existence of substructures and combinations of substructures.

**Fig. 6:**
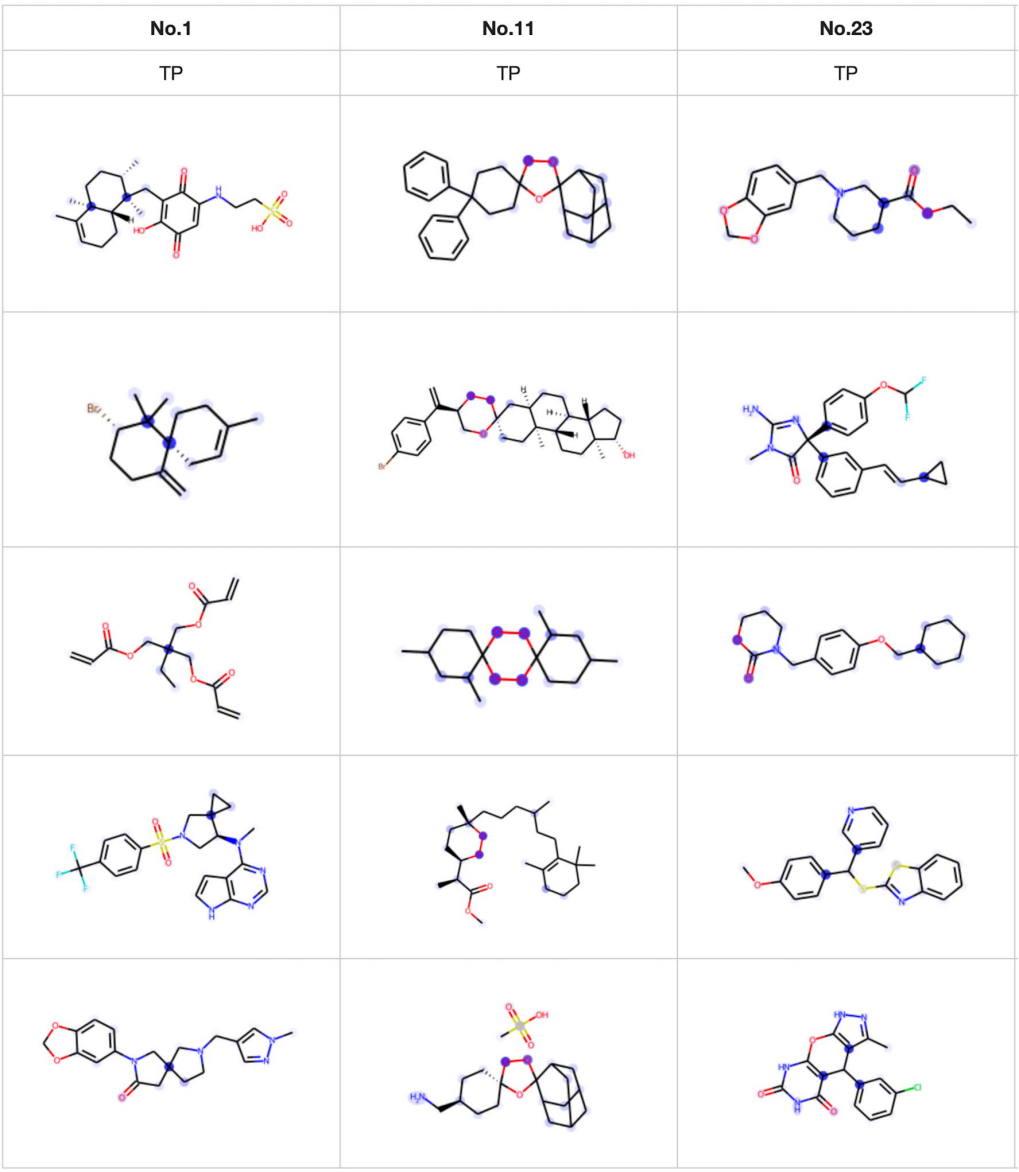
Visualization of the substructure prediction results

Next, we investigated the ability of the attention mechanism to interpret the prediction results by visualizing the attention matrix in the decoder-like module. We visualized only true positive (TP) samples (positive samples that were predicted correctly). The visualization results of the model that achieved the best average F1 value (six encoder layers and one decoder layer) are shown in Fig. 6. We visualize the results of 3 substructures in Fig. 6; the visualizations of the other substructures show the same trends as these 3 substructures. More TP results corresponding to each substructure can be found at [34]. According to Fig. 6, for No. 1, the attention mechanism identifies only part of the atoms in the target instead of all the atoms included in the target substructure. For substructure No. 11, the attention mechanism identifies only O-O in the target and does not identify the single O. This result shows that the attention mechanism does not identify the atoms in the substructures that are similar to the target. For substructure No. 23, even molecules that contain only cCc are identified as positive, and the attention mechanism identifies both CC(C)C and cCc. This result shows that the attention mechanism can identify atoms in all composition substructures related to the target.

**Fig. 7:**
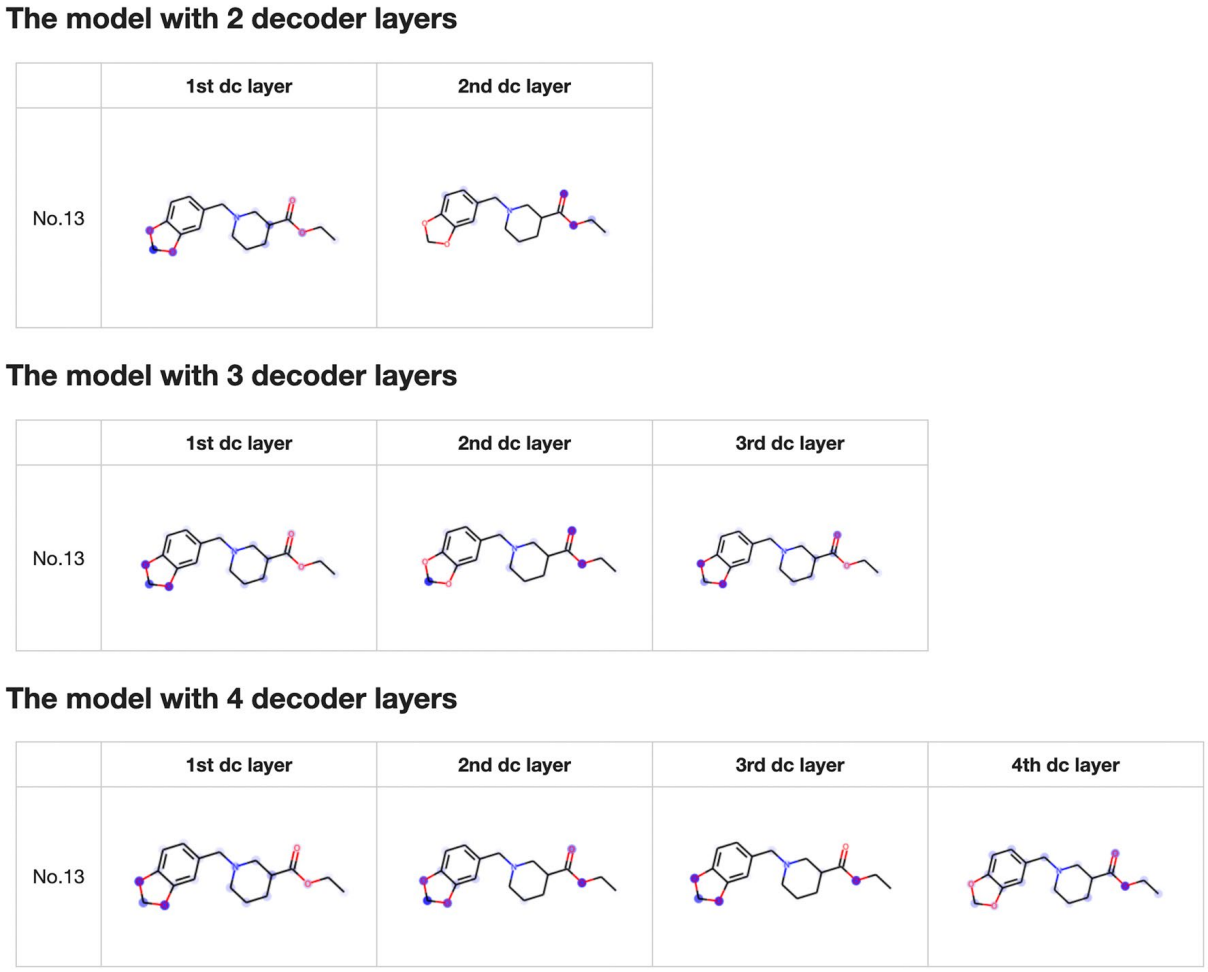
Visualization of the attention in each decoder layer for models with two, three, and four decoder layers

**Fig. 8:**
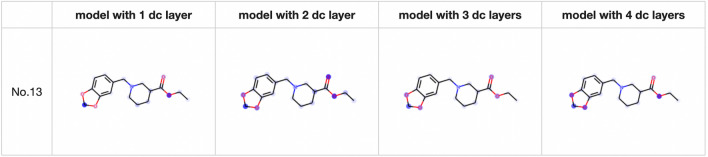
Visualization of the summed attention matrices

Figure 6 shows that the attention mechanism clearly identifies several atoms contained in the target substructures. To investigate the role of the attention mechanism in multiple decoder layers, we visualized models with two, three, and four decoder layers. Figure 7 shows the visualization results for each individual decoder layer. When the model has multiple decoder layers, the attention mechanism in each decoder layer can identify atoms related to the target substructure, which inspired us to visualize the sum of the attention mechanisms in all decoder layers. Figure 8 shows the visualization results of the summed attention, illustrating that models with different numbers of decoder layers can accurately identify atoms in the target substructures. Moreover, we used Eq. (5) to quantify the performance of the attention mechanisms with different numbers of decoder layers. We calculated Eqs. (5) and (6) for all targets and all samples. The average values of Eq. (5) for all 16 substructures corresponding to the four hyperparameter settings in Table 2 are 0.704, 0.602, 0.594, and 0.565, and the corresponding average values of Eq. (6) are 0.335, 0.204, 0.205, and 0.166. The attention mechanism in the model with one decoder layer tends to identify the target substructures with larger values and locates fewer atoms belonging to the target. From the above results, we can draw the following conclusions: (1) the attention mechanisms in the decoder layers can be used to interpret the prediction results; (2) for TP samples, the attention mechanisms identify only certain atoms instead of all atoms in the target substructures; and (3) for models with multiple decoder layers, the sum of the attention mechanisms in all decoder layers can identify the atoms related to the predictions.

### OD prediction results

**Table 4:** OD prediction results

	Macro F1	Micro F1
Proposed model	0.338	0.418
ADJ-only	0.333	0.417
DIST-only	0.316	0.398
MAT-attn	0.325	0.397
Simplified decoder	0.310	0.395
MAT model	0.264	0.351

The hyperparameter settings used in the OD prediction experiment and the optimal OD prediction settings are presented in Table 3. The results of our proposed model and the comparison model are shown in Table 4. The proposed model and the ADJ-only model achieve very similar results. Therefore, the attention values calculated by Eqs. (2) and (7) have similar effects on the results. We expected to introduce the 3D structure information of the molecules through *g*(*D*) in Eq. (2); however, the experimental results show that adding the distance information in this way does not enable the model to use the 3D structure information. This finding may be because there are relatively few samples, or the model itself may not have the ability to learn 3D structural information according to the distance matrix. The proposed model and the MAT-attn model obtain similar F1 results. Therefore, we conducted an approximate randomization test to verify whether the differences between these two results were meaningful. The p value was 0.009 when we compared the proposed and MAT-attn models.

The best average F1 value was achieved by the model with two decoder layers. Unlike the substructure prediction experiment, visualizing the attention of the first decoder layer shows that the attention mechanism tends to identify all atoms with similar values. This result may be caused by having relatively few samples. In fact, the same phenomenon was observed in the substructure prediction experiment when using the same number of samples as in the OD prediction experiment. However, even if we increase the number of samples to approximately 100,000 and perform the OD prediction experiment, there may still be a tendency for the first encoder layer attention mechanism to mark all atoms with similar values, it may be necessary to collect information about the whole molecule to predict the odor, as a result of the factors affecting the odor of a molecule being highly complex.

**Fig. 9:**
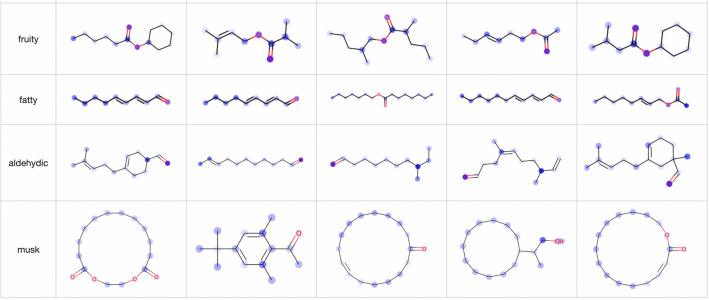
Visualization of the OD prediction results

Regarding attention visualization, we first visualized the attention of the model that achieved the best F1 value. We visualized the attention of the second decoder-like layer. More visualization results can be found at [34]. Nineteen ODs obtained F1 values greater than 0.45. To ensure that the visualization results are meaningful, we visualize only these 19 ODs. Figure 9 shows several visualization results of TP samples for ‘fruity’, ‘musk’, ‘aldehydic’ and ‘fatty’. For these four ODs, the attention mechanism tends to identify C(=O)O, carbon in a large ring, C=O and long carbon chains, respectively. However, for the remaining ODs, no obvious features are marked in the corresponding positive samples.

According to the substructure visualization experiment results, the attention mechanism annotates only certain atoms in the substructures instead of all related atoms. The atoms in each substructure are randomly annotated by the attention mechanism; that is, the marked atoms vary depending on the model initialization. To determine the substructures associated with the ODs, we repeatedly trained the models with the same hyperparameter settings and visualized the atoms that were frequently annotated by the attention mechanisms in the different models. Specifically, we trained the models with the same hyperparameters 100 times and created a counter for each atom in each molecule in the samples. For each model, we then identified the top *k* atoms in a given molecule with the largest attention values and increased the counters corresponding to these *k* atoms by 1. Finally, we determined the atoms with counter values greater than *n*. We visualized the attention mechanisms of 100 models with $k=5$ and $n=50$.

**Fig. 10:**
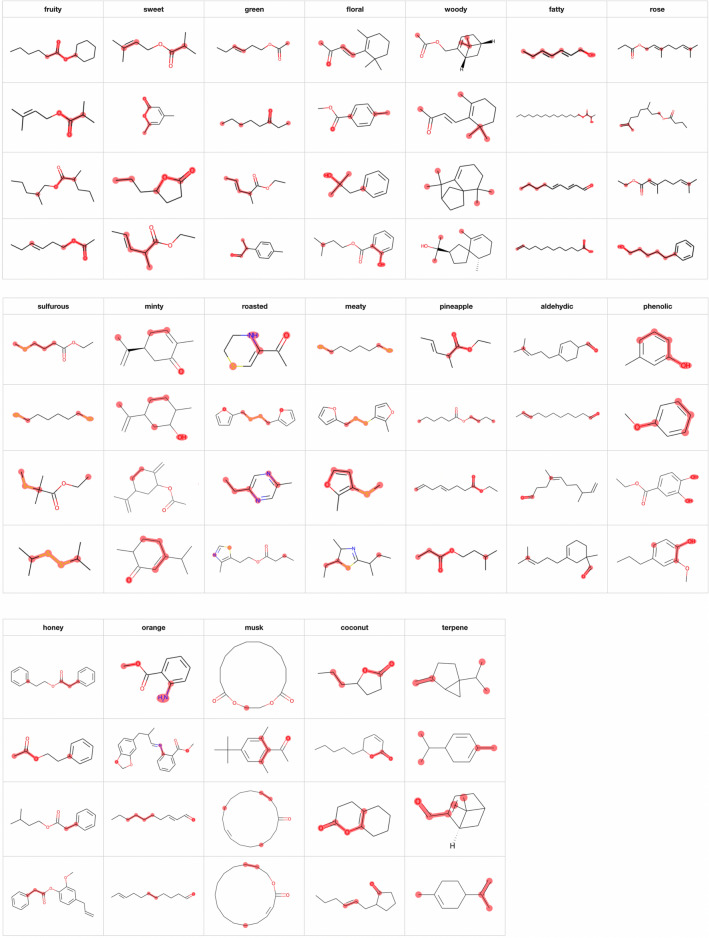
Visualization results of TP samples in the OD prediction experiment when $k=5, n=50$

**Fig. 11:**
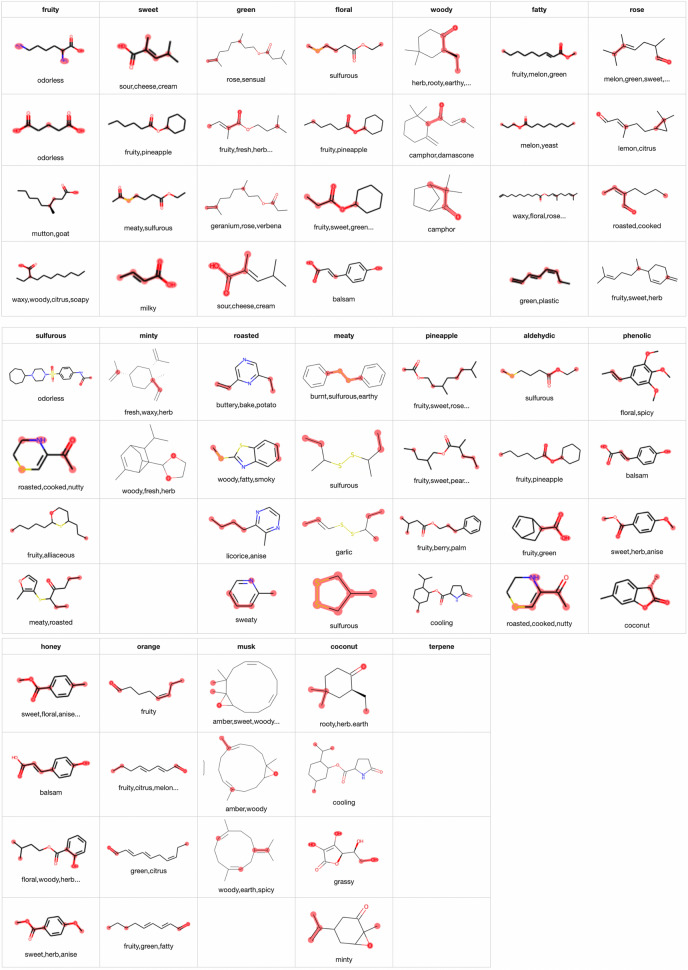
Visualization results of TN samples in the OD prediction experiment when $k=5, n=50$

**Table 5:** Summary of visualization results

	p	F1	TP	Substructure constraint	TN	Summary features
Fruity	242	0.636	Mainly annotates C(=O)O	C(=O)O and no atoms of C(=O)O in a ring.	Mainly C(=O)OH	C(=O)O
Sweet	208	0.509	Multiple structures, including C(=O)O	C(=O)O	Mainly C(=O)OH	Multiple structures, including C(=O)O
Green	189	0.520	C=C, C=O	C=O, C=C, CC(C)C and none of atoms in a ring	CC(=C)C	C=O and C=C without CC(=C)C
Floral	147	0.541	Multiple substructures, including C(=O)O, C(=O), C with 3 carbon neighbors, c1ccccc1	c1ccccc1C(=O)O or ‘A$\sim$A($\sim$A)$\sim$A’	No obvious features	Multiple substructures, including C(=O)O, C(=O), C with 3 carbon neighbors, c1ccccc1
Woody	107	0.517	CC(C)(C)C and three atoms of CC(C)(C)C in a ring	CC(C)(C)C and three atoms of CC(C)(C)C in a ring	Only 3 molecules, and these 3 samples are labeled by ODs such as ‘camphor’ and ‘earthy’	CC(C)(C)C and three atoms of CC(C)(C)C in a ring
Fatty	78	0.475	Carbon chain, C=O, -OH	’C$\sim$C$\sim$C$\sim$C$\sim$C $\sim$C$\sim$C$\sim$C’, with each C having only two heavy neighbors	Tends to mark C(=O)O vaguely	Long carbon chain
Rose	53	0.503	CC=C(C)C	CC=C(C)C’, c1ccccc1CCCC	C=O at the end	CC=C(C)C without C=O at the end
Sulfurous	43	0.709	S	S	S=O	S but not S=O
Minty	32	0.466	CC(=C)C1CCCCC1	CC(=C)C1CCCCC1	Only 2 molecules, and they are labeled by ODs such as ‘fresh’ and ‘herb’	CC(=C)C1CCCCC1
Roasted	36	0.470	’[n,s,o]’	’[n,s,o]’	Sometimes marks other atoms instead of ’[n]’	Substructures related ’[n,s,o]’
Meaty	36	0.591	’[SH]’, S	’[SH]’, SS	Tends to mark atoms on both sides of SS instead of SS	’[SH]’, SS and some neighboring substructures
Pineapple	28	0.467	C(=O)O	C(=O)O and no atoms of C(=O)O in a ring	Does not mark C(=O)O	May be a substructure containing C(=O)O
Aldehydic	22	0.462	C=O, C=C	C=O	Mainly C(=O)O	C(=O) but not C(=O)O
Phenolic	24	0.484	c1ccccc1O	c1ccccc1O	Tends to mark atoms whose neighbor is an aromatic carbon	c1ccccc1O
Honey	26	0.453	c1ccccc1 and C(=O)O	c1ccccc1 and C(=O)O	Marks c1ccccc1O vaguely	Both c1ccccc1 and C(=O)O exist in molecule
Orange	29	0.513	Carbon chain with C=O at the end	’C$\sim$C$\sim$C$\sim$C$\sim$C $\sim$C=O’, and none of the atoms are in a ring or have more than 3 heavy neighbors	Most samples are labeled as ‘fruity’ or ‘citrus’	Carbon chain with C=O at the end
Musk	11	0.493	Ring with more than 10 atoms	Ring with more than 10 atoms	Too few samples to summarize	Ring with more than 10 atoms.
Coconut	18	0.481	C(=O), with C in a ring	C(=O), with C in a ring	Tends to mark the end atoms that connected to a carbon in a ring	C(=O), with C in a ring and O at the end
Terpene	9	0.533	Too few samples	None	Too few samples	Too few samples

Since we considered 100 models, when we visualized the TP and TN (true negative samples, e.g., negative samples that were predicted correctly) samples, we chose positive samples that 90 of the 100 models predicted ODs as positive and negative samples that 85 models predicted as negative. Figure 10 shows the partial results of the TP samples of 19 ODs. In Fig. 10, because *k* is limited to five atoms, the ‘fatty’ and ‘musk’ visualization results are not as good as those in Fig. 9. For the other ODs, we can observe some clear features. According to the TP sample visualization results, we attempted to summarize the feature substructures for each OD, and the summary results are shown in the 4th column of Table 5. The number of positive samples in the test set corresponding to the 19 ODs is shown in the second column of Table 5. (We note that an OD corresponds to multiple feature substructures, and we summarize the features that appear most frequently in the visualization results.) In this experiment, we also visualized TN samples; in particular, we visualized only TN samples containing the feature substructures observed in the TP samples. The feature substructures used to screen TN samples are shown in the 5th column of Table 5, and several visualization results are shown in Fig. 11 (the corresponding OD labels of the molecule are also displayed). The features of the TN samples are summarized in the 6th column of Table 5. The TN sample visualizations show some interesting results. For example, ‘woody’ has only three TN samples under the corresponding substructure constraints; however, the OD labels of these three samples are ODs such as ‘camphor’ and ‘earthy’ somewhat similar to ‘woody’. Moreover, for ‘pineapple’, the TP sample visualization results show that C(=O)O is frequently annotated. However, for TN samples, the attention mechanism does not identify C(=O)O and instead annotates the neighbor of the C(=O)O substructure; therefore, we speculate that the region surrounding C(=O)O is also related to ‘pineapple’. Finally, according to the features of the TP and TN samples, we drew some conclusions about the feature substructures of the 19 ODs, as shown in the last column of Table 5.

Regarding the OD dataset, the amount of data is relatively small when using a neural network to predict ODs, and the consistency among the OD labels may also be an issue. For example, ‘fruity’ is a comprehensive odor, and molecules that are labeled ‘berry’, ‘apple’, etc., can be seen as ‘fruity’. However, in the collected data, some molecules that were labeled with fruit-like odors, such as ‘berry’, had OD labels that did not contain ‘fruity’. When we identified molecules containing fruit-like ODs (‘fruity’, ‘citrus’, ‘berry’, ‘apple’, ‘pineapple’, ‘orange’, ‘pear’, ‘melon’, ‘banana’, ‘lemon’, ‘coconut’, ‘peach’, ‘apricot’, ‘cherry’, ‘grape’, ‘grapefruit’, ‘plum’, ‘bergamot’, ‘hawthorn’, ‘jam’, ‘mandarin’, and ‘currant’) with the label ‘fruity’, the number of positive samples increased from 1334 to 1853, and the F1 value increased from 0.636 to 0.744. In addition, Chacko et al. [15] inferred the two ODs ‘sweet’ and ‘musky’ with the dataset in [35], which contains 480 samples, and achieved an F1 value of 0.81 for ‘sweet.’ We also trained and predicted ‘sweet’ odors with the dataset described in [35]. However, the small sample size led to unstable results, with the F1 value ranging from 0.71 to 0.91. Furthermore, we attempted to train the model with the dataset of 4462 samples used in this study and employed the dataset in [35] as the test set. The resulting F1 value was 0.69, which is higher than that in Table 5. We believe that this result is caused by the quality of the datasets. The dataset in [35] was constructed by 55 people scoring the odor for each odorant, and we took the average score assigned by these 55 individuals as the odorant label. The dataset used in this study was labeled by different people, and the labeling standards may vary from person to person. The F1 score of ‘sweet’ was approximately 0.50 with our datasets. This result may be influenced by the small number of samples and the lack of consistency in the labels across the large amount of collected data.

## Conclusion

In this study, we used a machine learning approach to investigate the relationship between molecular structure and odor. We first built a Transformer model to predict the molecular properties and interpret the prediction results. We modified the attention calculation in the encoder based on the MAT model and used a decoder-like module to interpret related substructures associated with ODs. We applied the proposed model to predict substructures in molecules and investigated the role of the attention mechanisms in the decoder layers. The results show that when we have a sufficient amount of samples, the attention mechanisms can identify some, but not all, of the atoms in the target substructures. This result demonstrates that the prediction results can be interpreted by visualizing the attention mechanism. Finally, we predicted 98 ODs with the proposed model and summarized the substructures associated with the 19 ODs by visualizing the attention mechanism. With additional odor labeling data, we expect to obtain better F1 results and clearer attention visualization results, thereby enabling a better understanding of the relationship between molecular structure and odor.

## Data Availability

The code used to collect data from The Good Scents Company, the experimental code, and attention visualization results are provided at https://github.com/zhenghah/0607.
